# Source Symbol Purging-Based Distributed Conditional Arithmetic Coding

**DOI:** 10.3390/e23080983

**Published:** 2021-07-30

**Authors:** Jingjian Li, Wei Wang, Hong Mo, Mengting Zhao, Jianhua Chen

**Affiliations:** School of Information Science and Engineering, Yunnan University, Kunming 650106, China; li_jingjian@mail.ynu.edu.cn (J.L.); weiwang@mail.ynu.edu.cn (W.W.); hongmo@mail.ynu.edu.cn (H.M.); justt@mail.ynu.edu.cn (M.Z.)

**Keywords:** distributed source coding, arithmetic coding, context model, purging

## Abstract

A distributed arithmetic coding algorithm based on source symbol purging and using the context model is proposed to solve the asymmetric Slepian–Wolf problem. The proposed scheme is to make better use of both the correlation between adjacent symbols in the source sequence and the correlation between the corresponding symbols of the source and the side information sequences to improve the coding performance of the source. Since the encoder purges a part of symbols from the source sequence, a shorter codeword length can be obtained. Those purged symbols are still used as the context of the subsequent symbols to be encoded. An improved calculation method for the posterior probability is also proposed based on the purging feature, such that the decoder can utilize the correlation within the source sequence to improve the decoding performance. In addition, this scheme achieves better error performance at the decoder by adding a forbidden symbol in the encoding process. The simulation results show that the encoding complexity and the minimum code rate required for lossless decoding are lower than that of the traditional distributed arithmetic coding. When the internal correlation strength of the source is strong, compared with other DSC schemes, the proposed scheme exhibits a better decoding performance under the same code rate.

## 1. Introduction

Distributed Source Coding (DSC) is a non-traditional data compression architecture that independently encodes multiple sources and jointly decodes them. It is different from traditional multi-source joint coding schemes, which require encoders to communicate with each other for collaborative working. DSC requires two (or more) related sources to be independently encoded, and then jointly decoded. DSC is based on two important theorems: the first is the Slepian–Wolf theorem [1], which proves that when data is losslessly compressed, independent coding and joint coding of related sources are equally effective; the second is the Wyner–Ziv theorem [2], which establishes the foundation for lossy DSC schemes. Today, DSC has led to achievements in many practical applications, such as: distributed video coding [3], hyperspectral image compression [4], multi-view video coding [5], wireless sensor network communication [6], image authentication [7], biometric information recognition [8] and so on.

Considering the following application scenario when two correlated wireless sensor nodes (i.e., sources *X* and *Y*) sending data to a base station (the receiver), as long as the code rates $R_{Y}⩾H\left(Y\right)$ and $R_{X}⩾H\left(X|Y\right)$ are met after *Y* and *X* are encoded, lossless compression can be achieved. Obviously, source *Y* can be encoded using any traditional entropy coder. In the case that *X* and *Y* can communicate with each other, it is very simple for source *X* to be encoded at a rate $R_{X}⩾H\left(X|Y\right)$ with an existing source coding scheme. However, communication between encoders consumes computing resources and the energy of the sensor nodes. Fortunately, the Slepian–Wolf theory guarantees that even no communication established between the two nodes, source *X* can also be encoded at a rate R(X) with $H\left(X\right)>R_{X}⩾H\left(X|Y\right)$ and be losslessly decoded at the decoder although the encoder for source *X* knows nothing about the source *Y* (hence the distributed source coding). This is the asymmetric Slepian–Wolf problem that this work focuses on. In this scenario, source *Y* is regarded as the side information which is encoded using a traditional source code at a rate $R_{Y}⩾H\left(Y\right)$. However, the coding scheme for source *X* is quite different, that is why the word ’asymmetric’ is used. Therefore, designing a method that enables *X* to be encoded at a rate R(X) less than H(X) without knowing the characteristics of *Y* and how to use the correlation between *X* and *Y* to losslessly recover *X* during the decoding process are the research goals of asymmetric Slepian–Wolf coding [9,10]. Apparently, the coding of *Y* is not the research focus of the asymmetric Slepian–Wolf problem; *Y* can usually be encoded by any lossless coding technology.

Although the coding theory of DSC was proposed long ago, it was not until the 21st century, when the important DSC scheme based on syndromes (DISCUS) was proposed for the asymmetric Slepian–Wolf problem in [11], that people carried out extensive research on specific coding schemes of DSC. After this, some DSC schemes based on channel coding have been proposed successively, such as the implementation of distributed source coding using Turbo codes [12], LDPC codes [13] and Polar codes [14]. The basic idea of DSC schemes based on channel codes is that the encoder only needs to send the syndromes or parity bits generated from the source sequence *X* to the decoder without the knowledge about *Y*, and the number of bits required to transmit the syndromes will be less than the number of bits required to directly transmit *X* using traditional source codes, thereby achieving further compression of *X*. The essence of the decoding process is that the decoder uses the received syndromes to ‘correct’ the side information sequence *Y* (the side information sequence *Y* can be regarded as an erroneous version of *X*). Coding methods of DSC schemes based on channel codes are apparently indifferent with the statistical characteristics of the source. This means that DSC schemes based on channel codes can only use the correlation between the sources, but cannot utilize the internal correlation among symbols of source *X*. Furthermore, DSC schemes based on channel codes can only exhibit better performances when coding longer blocks of source symbols.

In addition to DSC schemes based on channel codes, many scholars have also carried out research on DSC based on source codes. The DSC scheme based on arithmetic coding is the most popular method. Distributed arithmetic coding (DAC) [15] makes use of the characteristics of arithmetic coding that can freely adjust the encoding probability distribution of symbols during the encoding process; it ensures a shorter codelength by purposely expanding the probability interval of each symbol. However, the probability intervals during the coding will be overlapped after the probability interval of each symbol is expanded, which may cause the decoding ambiguity because the received codewords may fall into the overlapping areas during decoding. The decoder needs to create a decoding tree to save all possible decoding results, and use the maximum a posterior (MAP) decoding algorithm with the assistance of a side information sequence to find the decoding sequence with the largest cumulative product of posterior probabilities. Compared with DSC schemes based on channel codes, DAC can better adapt to non-stationary sources, and the coding performance for smaller data blocks will be better [9]. After DAC was proposed, a series of DSC schemes similar to DAC, which are based on expanding the probability intervals, were also successively proposed. In [16], a Slepian–Wolf code which is a quasi-arithmetic code based on coding interval overlapping is described, and this scheme is also applied to a distributed coding platform. Two non-binary DAC compression schemes, multi-interval DAC and Huffman coded DAC, are proposed in [17], which extend DAC from the compression of binary sources to the compression of non-binary sources. In [18], another DAC scheme was proposed which makes the encoder imitate the operation of the decoder and put ambiguous symbols in a list. If the decoder detects that the decoding has failed (e.g., by using a cyclic redundancy check code), the decoder can request the encoder to send the stored ambiguous symbols through a feedback channel. In [19], the DAC scheme was extended to a scenario of noisy communication, and introduced a ‘forbidden symbol’ to DAC; it is referred to as DJSCAC. This is a very convenient way to improve decoding error performance. On the basis of DAC, the scheme adds a non-coding interval into the original probability intervals, the non-coding interval does not correspond to any symbol that can be practically encoded. The decoder determines whether the codeword is wrong by judging whether the ‘forbidden symbol’ is obtained during the decoding process. However, when a DSC scheme based on expanding probability intervals encodes a biased source (the probability of one of the encoded symbol is relatively large), the large probability will make the expandable range of the probability interval very limited. This problem limits the potential compression gain that DSC schemes based on expanding probability intervals can obtain when the source has a highly skewed probability distribution $p0=p\left(x_{i}=0\right)\;or\;p1=p\left(x_{i}=1\right)\gg 0.5$, especially when a context model is used.

Another idea to implement the DSC scheme based on arithmetic coding is to puncture the output bitstream of the arithmetic encoder. This is a very convenient solution to obtain the additional compression gain, which will not be affected by the skewed probability distribution of the source. A DSC scheme using the punctured quasi-arithmetic code was proposed in [20]. Firstly, the quasi-arithmetic coding is used to encode the source to obtain the code stream, and then the bitstream is punctured (deletion of some bits in the bitstream) to further reduce the bit rate. A similar scheme by puncturing codewords is also proposed in [21]. This scheme uses arithmetic coding based on context models to encode the source symbols, and then the output bitstream is punctured to further reduce the code rate; a hierarchical interleaving scheme is used to avoid early mistaken deletion of the correct decoding path from the decoding tree. A coupled distributed arithmetic coding scheme was described in [22], which is also a method to obtain additional compression gain by puncturing the codeword of arithmetic coding. This scheme divides the original binary source sequence into a pair of coupled streams before arithmetic coding, and the decoder recovers these streams to overcome the de-synchronization problem. As long as the two streams do not lose synchronization at the same time, the decoder can still successfully recover the source sequence. However, the price of using this coupled method is that the original correlation between adjacent symbols in the source sequence is broken. In addition, the punctured bits in the codeword cannot be directly recovered; the decoder needs to insert bits into the received bitstream to restore the original length of the codeword. Meanwhile, there is no correlation in the bitstream that can be used to assist the restoration of those discarded bits. The decoder needs to perform bit insertion operations on each received byte, thereby increasing the decoding complexity.

It is now well established from a variety of studies that an excellent arithmetic code-based DSC scheme needs to have the following characteristics at the same time: it should make full use of the correlation between adjacent symbols in the source and the correlation between the corresponding symbols of the sources [23]. While further expanding the compression capability of traditional arithmetic coding, it should also have a good bit error rate at the decoder. Taking into account the shortcomings of the DSC schemes that puncture the bitstream, the method of puncturing the codeword can be changed to puncturing the source sequence, that is, purging part of the symbols from the source sequence.

Based on the above analysis, a new DSC algorithm named source symbol purging-based distributed conditional arithmetic coding (SPDCAC) is proposed in this paper. However, unlike the previous puncture-based schemes in which the punctured bits are simply discarded, the purged symbols in the proposed scheme are not only extracted from the source sequence to further reduce the bit rate, but are also used as the context for the coding of the subsequent symbols. The advantage of this method is that the original correlation between adjacent symbols in the source sequence is preserved, and the additional compression gain obtained will not be affected by the skewed probability distribution of the source. Meanwhile, the decoder can still use the correlation between adjacent source symbols (internal correlation of the source) to restore those purged symbols more accurately during the decoding process. A better calculation method of the posterior probability for SPDCAC is also proposed. Compared with the traditional method in [15], the proposed method exhibits a better bit error rate performance in the decoding process. A forbidden symbol is also added to the coding probability distribution, which can expedite the error detection and further reduce the bit error rate at the decoder but not break the correlation between adjacent symbols within the source, while not significantly affecting the compression ratio. The simulation results show that SPDCAC exhibits an excellent coding performance.

The rest of this paper is arranged as follows: In Section 2, the SPDCAC encoding and decoding details are described. Section 3 introduces the posterior probability calculation method proposed for SPDCAC. The simulation results are presented in Section 4. Section 5 is the summary of this paper.

## 2. Source Symbol Purging-Based Distributed Conditional Arithmetic Coding

**Figure 1:**
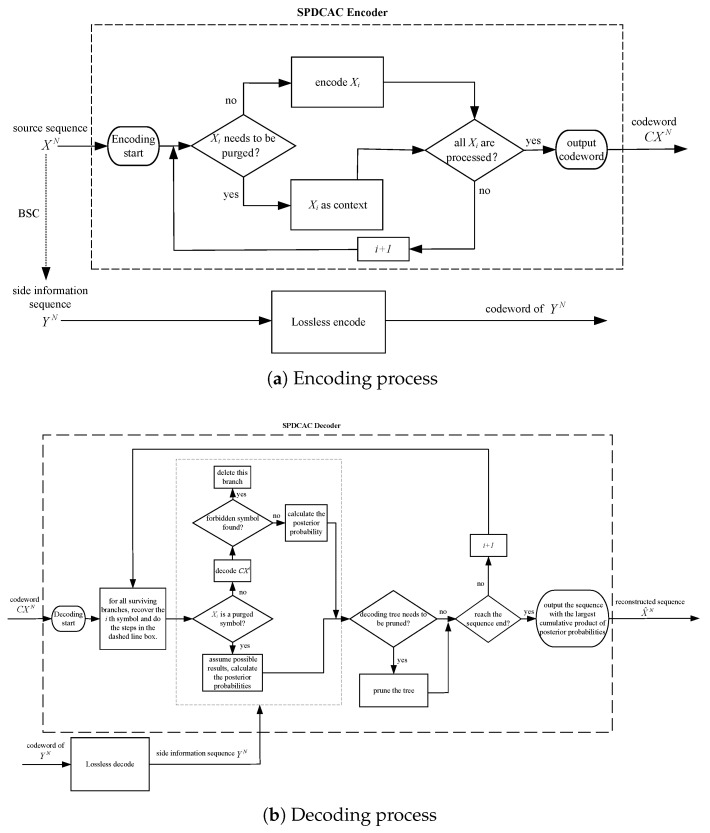
Block diagrams of the encoding and decoding processes of the proposed scheme.

The specific encoding and decoding processes of the proposed scheme are described in this section in detail. The block diagrams of the encoding and decoding processes are shown in Figure 1a,b, respectively.

### 2.1. SPDCAC Encoding Process

Suppose that the binary source sequence to be encoded is $X^{N}=\left(x_{1},x_{2},...,x_{N}\right)$. When the SPDCAC encoder based on a *k* th (k≥1)-order context model encodes this source sequence, the encoder needs to use *k* source symbols $\left(x_{i-1},x_{i-2},...,x_{i-k}\right)$ before the symbol $x_{i}$ to be encoded as the context condition, $\left(x_{i-1},x_{i-2},...,x_{i-k}\right)$ will be denoted as $x_{i-1}^{i-k}$, and the conditional probability: $P(x_{i}|x_{i-1}^{i-k})$ is used as the encoding probability. $P(x_{i}|x_{i-1}^{i-k})$ needs to be estimated by the counting statistics of the encoded symbols and dynamically updated during the encoding process. This means the underlying encoder of SPDCAC is a context-based adaptive arithmetic encoder.

**Figure 2:**
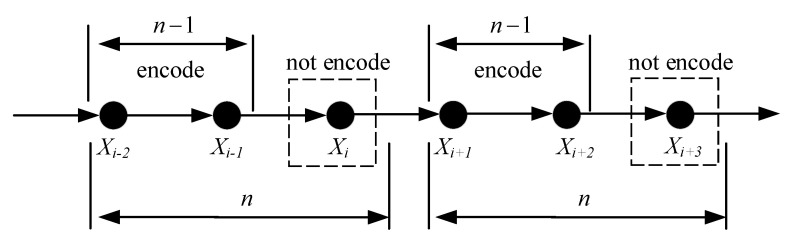
An example of the SPDCAC encoding process.

The encoder of SPDCAC only encodes a part of the symbols in the source sequence through the arithmetic encoder based on the context model. Other ‘purged’ symbols in the source sequence are not encoded and are only used as the context conditions for the coding of subsequent symbols. Figure 2 shows an example of the SPDCAC encoding process. The first n−1 symbols in every *n* source symbols need to be encoded, and the remaining ‘purged’ symbol together with those encoded symbols are used as the context conditions for the coding of following symbols (it can be regarded that the purging rate is 1/n). We use $CX^{i}$ to represent the codeword output by the SPDCAC encoder after encoding $X^{i}$. Since the underlying encoder of SPDCAC is an arithmetic encoder, as the encoding continues, bits are constantly added to the codeword, and the length of the codeword will continue to increase. It should be noted that if the encoder encodes the source symbol $x_{i}$ at moment *i*, but does not encode the source symbol $x_{i+1}$ at moment i+1, then the codeword $CX^{i+1}$ is the same as the codeword $CX^{i}$. When the encoder completes the encoding of the entire $X^{N}$ sequence, it outputs the codeword $CX^{N}$.

A forbidden symbol is considered during the encoding process of SPDCAC. The encoder of SPDCAC reserves a sub-interval that does not correspond to any valid source symbol in the encoding probability distribution intervals. This interval is related to a forbidden symbol that is not encoded. If there is no error in the codeword, the forbidden symbol should not be decoded during the decoding process. Otherwise, if the decoder finds that the codeword falls into the forbidden interval, it can be determined that the codeword is incorrect. Adding ‘forbidden symbols’ in the coding process will only increase the size of the alphabet, which will not break the internal correlation among the symbols in the source sequence. This approach can rapidly detect whether the codeword is wrong so that the decoder can terminate the corresponding branch in the decoding tree immediately to improve decoding performance.

**Figure 3:**
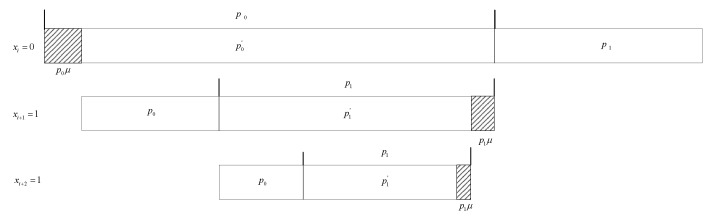
An example of SPDCAC coding interval adjustment process with a forbidden symbol.

As shown in Figure 3, in the probability distribution for a binary source, the forbidden sub-interval is added at the end of the probability intervals right beside the sub-interval of the symbol with the larger probability. The length of the forbidden sub-interval is of length μ which is only related to the larger probability. Let $p_{0}$ and $p_{1}$ be the original probabilities of the symbols 0 and 1, respectively. If $p_{0}$ is larger, it will become $p_{0}^{{}^{\prime }}$ after the forbidden sub-interval μ is added; otherwise, $p_{1}$ will become $p_{1}^{{}^{\prime }}$.

### 2.2. SPDCAC Decoding Process

**Figure 4:**
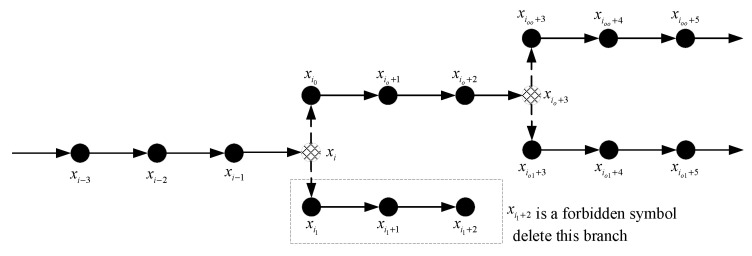
A part of the decoding tree where $x_{i}$ is a purged symbol.

The SPDCAC decoder needs to use the received codeword to reconstruct the original source sequence as accurately as possible with the help of a side information sequence, which is correlated to the source sequence. The initial probability distribution used by the decoder must be the same as that of the encoder. The conditional probability distributions in the context model at the decoder need to synchronously perform the same counting and updating operations as what is performed at the encoder. The decoder must know the purging rate of the encoder. In the actual coding process, the purging rate can be preset to be the same in the encoder and the decoder. In the encoding process, those purged symbols are not encoded, but they are used as context conditions for the encoding of subsequent symbols. Therefore, the SPDCAC decoder cannot recover those purged symbols by directly decoding the codeword, and the context probability model of the decoder does not count those symbols. This will let the SPDCAC decoder use an incorrect probability distribution to decode the received codeword, the decoded result must be wrong. If a decoding error occurs during the decoding process, due to the error propagation characteristics of arithmetic coding, the decoding error will continue to appear in the subsequent decoding process, making the decoding completely fail. In order to decode successfully, the SPDCAC decoder has to recover those purged symbols according to the purging rate of the encoder. For the recovering of an unencoded symbol, the decoder must make assumptions about all possible symbols in this position. This means the decoding ambiguity phenomenon occurs. In the process of decoding a binary sequence, whenever the decoding ambiguity takes place, two possible decoding results will appear, the decoder has to use a binary decoding tree to record this event, and two new decoding branches are extended from the original decoding branch based on these two results (a new branch is added to the decoding tree). The decoder stores all possible decoding branches of the binary decoding tree, where each path in the tree represents a possible decoding output sequence. Figure 4 shows a part of the decoding tree where $x_{i-1}$∼$x_{i-3}$ are unambiguously decoded symbols in one of the branches in the decoding tree, and these symbols are encoded by the context-based arithmetic encoder, so they can be directly decoded at the decoder. $x_{i}$ is a symbol that is ‘purged’ at the encoder. The decoder needs to assume two possible decoding results for $x_{i}$ at this position, and based on these two results, independent decoding processes on their respective decoding branches are performed. Let $x_{i}^{0}$ and $x_{i}^{1}$ be the two possible decoding results of $x_{i}$, the decoder can obtain two different decoding branches: $x_{i}^{0},x_{i+1}^{0},\cdots$ and $x_{i}^{1},x_{i+1}^{1},\cdots$. The decoding process of each decoding branch needs to be synchronized, which means that the decoder needs to simultaneously decode the symbols at: i,i+1,⋯ in two different branches.

The specific decoding process can be described as follows:

1. Decoding starts;
2. Determine whether the current decoding position *i* is less than or equal to the sequence length *N*; if so, perform the operation:
  (a) Synchronously decode the *i* th symbol of all decoding branches that are still alive in the decoding tree:
    - If the symbol that needs to be decoded at the current position *i* is encoded at the encoder, the decoder can use the symbols at positions i−1∼i−k in the current decoding sequence as the context conditions of the current decoding symbol and perform decoding to obtain a clear decoding result. If the decoding result is a forbidden symbol, the current decoding branch is deleted. If the decoding result is a symbol 0 or 1, the decoder calculates the posterior probability $\lambda_{i}$ of the decoding symbol and the cumulative product of posterior probabilities $\Lambda_{i}$ of the current decoding branch with the help of the side information sequence, and then the decoding tree continues to extend forward.
    - If the symbol that needs to be decoded at the current position *i* is a symbol that is not encoded at the encoder, the decoder needs to assume two possible results at this position: 0 and 1. In this way, a new decoding branch is added to the decoding tree. Then, the posterior probabilities $\lambda_{i}^{0}$ and $\lambda_{i}^{1}$ of the two hypothetical symbols are calculated. Similarly, the cumulative product of posterior probabilities $\Lambda_{i}^{0}$ and $\Lambda_{i}^{1}$ of the two decoding branches are also calculated. Both decoding branches will continue to extend one step forward.
  (b) If the number of paths in the decoding tree reaches the predetermined upper bound, the decoding tree will be pruned and half of the decoding paths with the smallest cumulative product of posterior probabilities are deleted.
3. In the end, the decoder outputs the decoded sequence related to the path with the largest cumulative product of posterior probabilities as the final decoding sequence.

## 3. The Calculation of the Posterior Probability

According to the theory of the context-based arithmetic coding, only if the decoder uses the same conditional probability distributions in the context model and follows the same updating rules as that of the encoder, can the source sequence $X^{N}$ be recovered correctly using the received codeword $CX^{N}$. Since only part of the symbols in $X^{N}$ are encoded by an arithmetic encoder based on the context model, the remaining purged symbols are not encoded (they are still used as the context); it is impossible for the decoder to know the correct updating rules for those conditional probability distributions in the context model. Without the correctly updated context model, the decoded sequence from $CX^{N}$ is erroneous and irrelevant to the original source sequence $X^{N}$. However, with the help of the side information sequence, for moment *i*, we can utilize the correlation between $X^{i-1}$ and $Y^{i-1}$ to estimate the source sequence $X^{i-1}$ as accurately as possible such that the context model could be updated properly, and the correct symbol $X_{i}$ could be decoded from $CX^{i}$. To this end, since we have the side information sequence $Y^{N}$, we can calculate the cumulative product of posterior probabilities of all estimated ${\hat{X}}^{i-1}$ sequences, and use the one with the largest cumulative product of posterior probabilities as the estimate of $X^{i-1}$.

In DAC research [15], Formula (1) is usually used to calculate the cumulative product of posterior probabilities of each decoding path from moment 1 to *N*

(1) $$\Lambda =P\left({\hat{X}}^{N}|Y^{N},CX^{N}\right)=\prod_{i=1}^{N}P\left({\hat{X}}_{i}={\hat{x}}_{i}|Y_{i}=y_{i},CX^{i}\right)$$

where ${\hat{X}}^{N}$ represents one of the decoded symbol sequences in the decoding tree from moment 1 to *N*, $Y^{N}$ represents the corresponding sequence of side information *Y*, $CX^{N}$ is the SPDCAC encoder output codeword after encoding $X^{N}$, and $CX^{i}$ is the codeword received up to the moment *i*. A virtual binary symmetric channel (BSC) is usually used in the DSC research to describe the correlation between any $X_{i}$ and $Y_{i}$ pair [24], the transition probability $P(y_{i}|x_{i})$ of the channel measures the correlation between the two sources. A small transition probability represents the strong correlation between the $X_{i}$ and $Y_{i}$ pairs. Since BSC is a memoryless channel, the side information symbol $Y_{i}$ is considered only related to the corresponding original source symbol $X_{i}$, but not related to $X_{i-1}$, $X_{i+1}$, $Y_{i-1}$ and $Y_{i+1}$. However, this posterior probability calculation method only considers the correlation between the symbols of two sources at moment *i*, but does not consider the situation of sources with memory. In the decoding process of SPDCAC, the correlation between adjacent symbols within the source sequence should also be used as an important part to determine whether the decoding result is correct. The conditional probability in the context model can be added to the posterior probability calculation as a means of using this correlation.

Since the side information sequence $y^{N}$ is known during the calculation of the posterior probability, the joint probability $P(Y^{N}=y^{N})$ is considered as the prior probability of the side information sequence, and is also known and is a constant. $CX^{N}$ is the unique codeword received by the decoder; it is fixed for all decoding branches in the decoding tree. Therefore, the probability $P(CX^{N})$ is also considered as the prior probability of $CX^{N}$ during the decoding process. From the perspective of the decoder, before the correct source sequence $X^{N}$ is found, the codeword $CX^{N}$ and the source sequence $X^{N}$ are independent because the decoder does not know which path in the decoding tree corresponds to the correct source sequence $X^{N}$. Since the side information sequence $Y^{N}$ is only correlated with $X^{N}$, the codeword $CX^{N}$ is actually independent of the side information $Y^{N}$ at the decoder side until the correct source sequence $X^{N}$ is estimated and the context model is updated properly. Which means $P\left(Y^{N}=y^{N},CX^{N}\right)=P\left(Y^{N}=y^{N}\right)P\left(CX^{N}\right)$. In this way, multiplying $P(Y^{N}=y^{N},CX^{N})$ to both sides of (1), the equality also holds.

(2) $$\begin{array}{cc} \tilde{\Lambda } & =P({\hat{X}}^{N}={\hat{x}}^{N},Y^{N}=y^{N},CX^{N})\\ \; & =P\left({\hat{X}}^{N}={\hat{x}}^{N}|Y^{N}=y^{N},CX^{N}\right)\cdot P\left(Y^{N}=y^{N},CX^{N}\right)=\prod_{i=1}^{N}P\left({\hat{X}}_{i}={\hat{x}}_{i},Y_{i}=y_{i},CX^{i}\right) \end{array}$$

The above $\tilde{\Lambda }$ can be regarded as an estimate of Λ. At any moment *i* during the decoding process, the rightmost term of Equation (2) can be rewritten as $P({\hat{X}}^{i}={\hat{x}}^{i},Y^{i}=y^{i},CX^{i})$ and decomposed recursively according to Equation (3) which expresses the relationships that exist among the decoded sequence ${\hat{X}}^{i}$, the side information sequence $Y^{i}$, and the codeword sequence $CX^{i}$:

(3) $$\begin{array}{cc} \; & P({\hat{X}}^{i}=x^{i},Y^{i}=y^{i},CX^{i})\\ \; & =P({\hat{X}}_{i}=x_{i}|{\hat{X}}^{i-1}={\hat{x}}^{i-1},Y^{i}=y^{i},CX^{i})\\ \; & \cdot P(Y_{i}=y_{i}|{\hat{X}}^{i-1}=x^{i-1},Y^{i-1}=y^{i-1},CX^{i})\\ \; & \cdot P(X_{i-1}={\hat{x}}_{i-1}|{\hat{X}}^{i-2}={\hat{x}}^{i-2},Y^{i-1}=y^{i-1},CX^{i})\\ \; & \cdot P(Y_{i-1}=y_{i-1}|{\hat{X}}^{i-2}={\hat{x}}^{i-2},Y^{i-2}=y^{i-2},CX^{i})\\ \; & \cdots \\ \; & \cdot P({\hat{X}}_{2}={\hat{x}}_{2}|{\hat{X}}_{1}={\hat{x}}_{1},Y^{2}=y^{2},CX^{i})\\ \; & \cdot P(Y_{2}=y_{2}|{\hat{X}}_{1}={\hat{x}}_{1},Y_{1}=y_{1},CX^{i})\\ \; & \cdot P\left({\hat{X}}_{1}={\hat{x}}_{1}|Y_{1}=y_{1},CX^{i}\right)\cdot P\left(Y_{1}=y_{1},CX^{i}\right) \end{array}$$

As mentioned before that the relationship between $Y_{i}$ and $X_{i}$ is represented by a virtual BSC channel, and $Y_{i}$ is only related to $X_{i}$, and independent of $X_{i-1}$, $X_{i+1}$, $Y_{i-1}$ and $Y_{i+1}$, the probability $P(Y_{i}|{\hat{X}}^{i},Y^{i-1},CX^{i})$ can be represented as $P(Y_{i}|X_{i},CX^{i})$ in (3). Since the codeword $CX^{i}$ is independent of $Y_{i}$, $P(Y_{i}|{\hat{X}}_{i},CX^{i})$ can be further reduced to $P(Y_{i}|{\hat{X}}_{i})$.

In the encoding process, if the encoder uses $X_{i-1}^{i-k}$ as the context conditions to encode $X_{i}$, then, in the decoding process, the decoder must decode ${\hat{X}}_{i-1}^{i-k}$ before they can be used as the conditions to decode ${\hat{X}}_{i}$. Obviously, ${\hat{X}}_{i}$ is related to ${\hat{X}}_{i-1}^{i-k}$, but it is considered that ${\hat{X}}_{i}$ is not related to the previously decoded symbols ${\hat{X}}^{i-k-1}$. Similarly, according to the property of the BSC channel, ${\hat{X}}_{i}$ is not related to $Y^{i-1}$. Therefore, $P({\hat{X}}_{i}|{\hat{X}}^{i-1},Y^{i-1},{\hat{CX}}^{i})$ in (3) can be simplified to $P({\hat{X}}_{i}|{\hat{X}}_{i-k}^{i-1},CX^{i})$.

The posterior probability calculation method similar to (3) has been used in [21], that is $\lambda_{i}=logP\left({\hat{X}}_{i}|Y_{i}\right)+logP\left({\hat{X}}_{i}|{\hat{X}}_{i-1}\right)$, which not only calculates the probability of each ${\hat{X}}_{i}$ relative to the known $Y_{i}$, but also calculates the probability of each ${\hat{X}}_{i}$ appearing under the condition ${\hat{X}}_{i-1}$. This method utilizes the correlation among adjacent symbols in the source sequence to reduce the bit error rate of the decoding result. However, SPDCAC is a DSC scheme that purges the source sequence; those purged symbols are not encoded. The recovery method of the purged symbols should be different from that of other symbols. Therefore, the posterior probability calculation method of SPDCAC in different decoding cases should be considered.

In the actual encoding and decoding processes, the codeword $CX^{i}$ contains the information of the encoded symbols. In other words, when the context condition ${\hat{X}}_{i-k}^{i-1}$ is known, the codeword $CX^{i}$ must be decoded to a determined symbol ${\hat{X}}_{i}$. For $P({\hat{X}}_{i}|{\hat{X}}_{i-k}^{i-1},CX^{i})$, if both ${\hat{X}}_{i-k}^{i-1}$ and $CX^{i}$ are known, then there must be a determined decoding result ${\hat{X}}_{i}$; in this case $P({\hat{X}}_{i}|{\hat{X}}_{i-k}^{i-1},CX^{i})=1$. In the encoding process of SPDCAC, if a symbol $X_{i}$ is not encoded at moment *i*, the information of $X_{i}$ is not included in the codeword $CX^{i}$. In the decoding process at this moment, $CX^{i}$ cannot provide any information about ${\hat{X}}_{i}$, and it is necessary to make two alternative decoding attempts at this position. Therefore, at this moment, $P({\hat{X}}_{i}|{\hat{X}}_{i-k}^{i-1},CX^{i})\neq 1$, there should be $P\left({\hat{X}}_{i}|{\hat{X}}_{i-k}^{i-1},CX^{i}\right)=P\left({\hat{X}}_{i}|{\hat{X}}_{i-k}^{i-1}\right)$. However, if $X_{i}$ is an encoded symbol, regardless of the previous decoding process; it should be considered that both ${\hat{X}}_{i-k}^{i-1}$ and $CX^{i}$ are known at moment *i*, and we have $P({\hat{X}}_{i}|{\hat{X}}_{i-k}^{i-1},CX^{i})=1$.

In this way, the posterior probability calculation method of SPDCAC can be represented by:

(4) $$\begin{array}{cc} \tilde{\Lambda } & =P({\hat{X}}^{N}={\hat{x}}^{N},Y^{N}=y^{N},CX^{N})\\ \; & =\prod_{i=1}^{N}P\left(Y_{i}=y_{i}|{\hat{X}}_{i}={\hat{x}}_{i}\right)\cdot P\left({\hat{X}}_{i}={\hat{x}}_{i}|{\hat{X}}_{i-k}^{i-1}=x_{i-k}^{i-1},CX^{i}\right) \end{array}$$

The logarithmic form of (4) is:

(5) $$\begin{array}{cc} log\tilde{\Lambda } & =\log P({\hat{X}}^{N}=x^{N},Y^{N}=y^{N},CX^{N})\\ \; & =\sum_{i=1}^{N}\left(logP(Y_{i}=y_{i}|{\hat{X}}_{i}={\hat{x}}_{i})+logP({\hat{X}}_{i}={\hat{x}}_{i}|{\hat{X}}_{i-k}^{i-1}={\hat{x}}_{i-k}^{i-1},CX^{i})\right) \end{array}$$

where

(6) $$\begin{array}{c} P\left({\hat{X}}_{i}={\hat{x}}_{i}|{\hat{X}}_{i-k}^{i-1}={\hat{x}}_{i-k}^{i-1},CX^{i}\right)=\left\{\begin{array}{cc} 1 & x_{i}\;is\;encoded\\ P({\hat{X}}_{i}={\hat{x}}_{i}|{\hat{X}}_{i-k}^{i-1}={\hat{x}}_{i-k}^{i-1}) & x_{i}\;is\;purged \end{array}\right. \end{array}$$

This calculation method is only related to the BSC channel crossover probability and conditional probability distributions in the context model. It can not only use the correlation between the source sequence and the side information sequence, but can also selectively utilize the correlation among source symbols according to different decoding cases to ensure a better decoding BER (bit error rate) performance. It should be noted that DSC schemes based on expanding probability intervals such as DAC and DJSCAC cannot employ the proposed method, since, in these schemes, the time when the codeword falls into the overlapping area during the decoding process is not determined, the timing and number of occurrences of decoding ambiguity in different decoding paths are different. It is difficult to fairly compare the cumulative product of posterior probabilities in different paths, since the calculation of $P({\hat{X}}_{i}={\hat{x}}_{i}|{\hat{X}}_{i-k}^{i-1}={\hat{x}}_{i-k}^{i-1},CX^{i})$ depends on the occurrence of decoding ambiguity. If the decoder of DAC use (5) to calculate the posterior probability, the cumulative product of posterior probabilities of a decoding path with many ambiguities will be much smaller than that of another decoding path with a few ambiguities. Apparently, such a difference between the cumulative product of posterior probabilities is not caused by the different correlations between the corresponding decoding sequences and the side information sequence.

## 4. Simulation Results

This section presents simulation results to evaluate the performance of the proposed scheme. Since the SPDCAC encoder encodes the source sequence *X* with a code rate less than H(X), the SPDCAC decoder cannot directly decode the received codeword to restore *X*. Therefore, we investigate the encoding performance of SPDCAC and the minimum code rate required of SPDCAC to achieve lossless decoding with the help of a side information sequence that has a strong correlation with *X*. As the correlation strength between the source sequence and the side information sequence gradually weakens, errors in the decoded results are also gradually increasing. The decoding performance of SPDAC with the assistance of different side information is also our focus. Since the scheme proposed in this article needs to use the context model in the coding process, a randomly generated binary order-1 Markov source sequence of length *N* will be used as the source sequence *X*. The transition probability matrix of the Markov source is shown in Table 1, where the value of $P_{t}$ is positively correlated with the strength of the correlation between adjacent symbols in the source sequence. All schemes based on arithmetic coding in subsequent experiments will use the order-1 context model in the coding process.

The code rate unit of all DSC schemes is bit/symbol (b/s), which is obtained by dividing the number of bits of the codeword output from the encoder by the number of source symbols. The side information sequence *Y* is generated by sending the source sequence *X* through a virtual BSC. Every symbol in *Y* is transmitted to the decoder losslessly. The correlation between *X* and *Y* is controlled by the transition probability of BSC (the transition probability of BSC is $p_{c}$), and the conditional entropy H(X|Y) between *X* and *Y* is used to represent the correlation between them. $H\left(X|Y\right)=H\left(p_{c}\right)=-p_{c}log_{2}p_{c}-$$\left(1-p_{c}\right)log_{2}\left(1-p_{c}\right)$ [24]. A smaller H(X|Y) means there is a higher similarity between *X* and *Y*. The conditional entropy H(X|Y) was set to 0.1∼0.5 in experiments. Many DSC studies use BER as the performance evaluation index of decoding, which is used to indicate the ratio of the number of error bits to the total number of bits in the decoding result. It is also used to show the decoding performance in our experiments. In our experiment, the purging rates of the encoder and the decoder were preset to be the same. The interval expansion factor α of the DSC scheme based on expanding probability intervals was also preset to be the same in the encoder and the decoder. Due to the randomness of the generated source sequence, each result shown in the following figures and tables is the averaged value of $10^{3}$ experiments.

### 4.1. Performance of the Improved Posterior Probability Calculation Method

**Figure 5:**
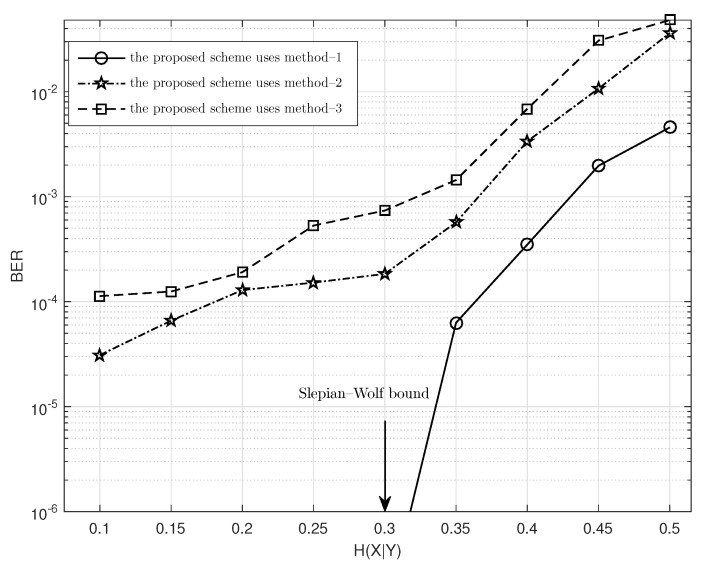
The BER performance of the proposed scheme using different posterior probability calculation methods.

The decoding performance of SPDCAC is closely related to the posterior probability calculation method used in decoding. Figure 5 shows the BER performance of SPDCAC using three different posterior probability calculation methods in the decoding process. After the encoder of SPDCAC encodes a binary Markov source ($P_{t}=0.9$) of length N=1024 at a rate about 0.3 b/s, the decoder of SPDCAC uses three different posterior probability calculation methods to decode the source sequence under different H(X|Y) (the maximum width of the decoding tree is limited to 256), and then, their respective BER performance results are obtained. The three calculation methods for the posterior probability are: 1. the method proposed in (5); 2. the method used in [21], that is $\lambda_{i}\;=\;logP\left({\hat{X}}_{i}|Y_{i}\right)+logP\left({\hat{X}}_{i}|{\hat{X}}_{i-1}\right)$; 3. the method used in DAC [15]. It can be seen from the figure that the application of the context model for the calculation of the posterior probability during the decoding process has a significant impact on the BER performance of the decoder. Both methods 1 and 2 use the context model in the posterior probability calculation, and their BER performances are significantly better than method 3 which does not employ the context model. Meanwhile, the BER performance of method 1 is far better than that of the other two. Especially when H(X|Y)<0.35, method 1 can obtain lossless decoding results, while the other two methods cannot.

The posterior probability calculation in [21] makes use of the conditional probability $P(X_{i}|X_{i-1})$, so it exhibits a better BER performance than the method in [15]. However, that scheme does not consider the different decoding cases in the decoding process (whether the decoding ambiguity appears). Those cases are taken into account in the proposed algorithm, where the conditional probability $P(X_{i}|X_{i-1})$ is only calculated at the positions where $x_{i}$ are purged. This can expand the gap between the values of the cumulative product of posterior probabilities of different decoding paths. The most likely correct decoding path will quickly accumulate to a greater posterior probability. Therefore, the algorithm proposed in this paper is more effective than the scheme in [21] in terms of BER performance.

### 4.2. The Required Minimum Code Rate for Lossless Decoding

For lossless decoding, the required minimum code rate (RMCR) of a DSC scheme can be used to present the coding efficiency of that DSC scheme. In this experiment, the RMCR of SPDCAC was compared with that of the traditional distributed arithmetic coding. However, due to a ‘forbidden symbol’ being added in the SPDCAC codec, we chose DJSCAC to compare with SPDCAC, and the results of arithmetic coding (AC) are provided for reference. We adjusted the value of $P_{t}$ to obtain binary Markov sources with different internal correlation strengths. The length of each sequence is 1024. In a DSC scheme based on expanding probability intervals, in order to obtain additional compression, the probability intervals of the encoding distribution need to be enlarged. However, if the probability of the current symbol is already large enough, the extent to which the probability can be enlarged is very limited, because the symbol probability cannot exceed 1. Based on the above analysis, in order to make DJSCAC obtains the largest possible compression, and the maximum possible coding probability interval of DJSCAC needs to be set by choosing the largest factor α(0≤α<1), which meets the inequality.

(7) (1+α)p≤(1−μ)

where *p* represents the larger probability in the binary conditional distribution used for the coding of the current symbol. It should be noted that in DJSCAC, the forbidden interval is located at the right end of probability intervals adjacent to the interval of symbol ‘1’, its length μ is set to 0.01. Similarly, the length of μ is set to 0.01 for SPDCAC.

Table 2 shows the RMCR of SPDCAC and DJSCAC coding different sources when H(X|Y)=0.1. From the table, we can find that the code rate of the arithmetic coding when encoding a source with a larger $P_{t}$ will be smaller than that when coding a source with a smaller $P_{t}$. This shows that arithmetic coding can effectively utilize the internal correlation of the source to reduce the code rate. All DSC schemes based on arithmetic coding also have this property—that is, as the internal correlation strength of the source increases, lossless decoding can be realized at a smaller code rate. The RMCR of DJSCAC will be smaller than SPDCAC only when coding the source with a weaker internal correlation ($P_{t}=0.6$). However, in other cases, the RMCR of SPDCAC is significantly smaller than that of DJSCAC. In particular, when coding a source with $P_{t}=0.9$, SPDCAC can save 50% of the code rate than AC, while DJSCAC can only save 29% of the code rate than AC. This phenomenon shows that the RMCR of SPDCAC is significantly better than that of DJSCAC when coding a source with a stronger internal correlation strength.

### 4.3. Decoding Performance

In order to evaluate the decoding performance of SPDCAC, the proposed scheme will be compared with several other DSC schemes. Those DSC schemes used for comparison with SPDCAC in this experiment are DJSCAC and the DSC scheme based on irregular LDPC code, respectively. The DSC scheme based on LDPC code use the approach proposed in [13]. After DSC schemes encoding the same source sequence at the same code rate, the same side information sequence is used to help the decoding of these schemes. Both DJSCAC and SPDCAC use the same forbidden interval μ=0.01. DJSCAC controls the code rate by adjusting the maximum admissible range of α in (7). During the coding process for each symbol, α is firstly calculated by (7), if its value exceeds a predetermined threshold, set α to this threshold; otherwise, α remains unchanged. In this experiment, the maximum width of the decoding tree in the MAP algorithm used by DJSCAC and SPDCAC is limited to 256.

**Figure 6:**
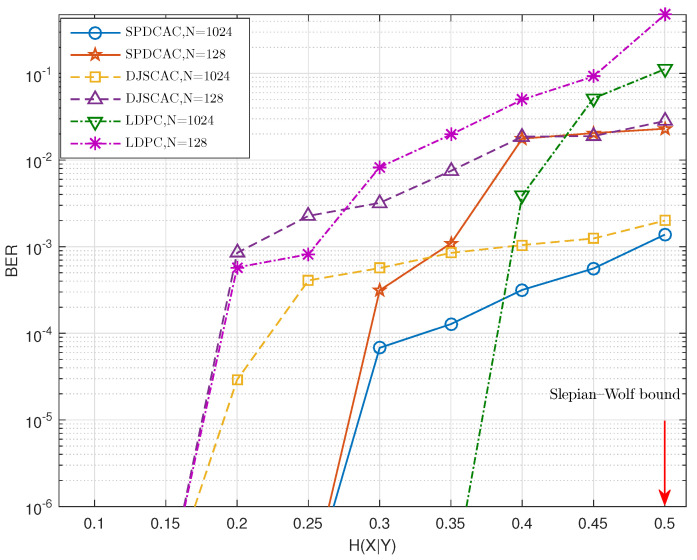
BER performance of different DSC schemes when encoding Markov sources ($P_{t}=0.8$) with various *N* at a fixed code rate of 0.5 bit/symbol.

**Figure 7:**
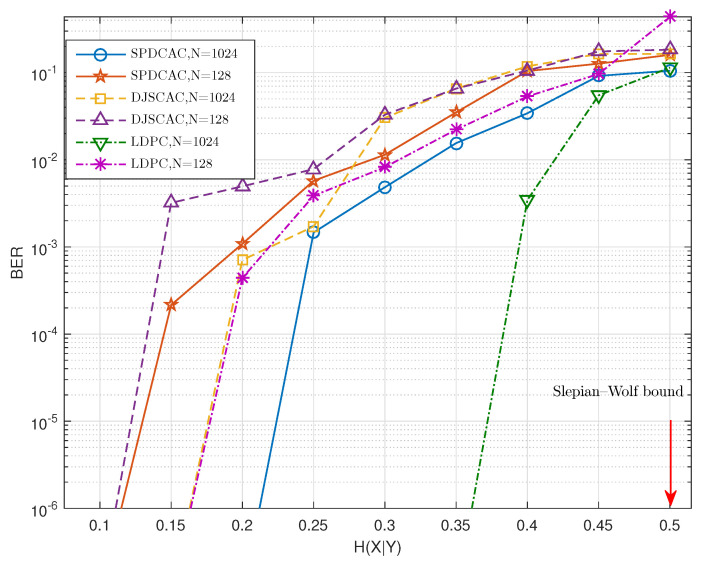
BER performance of different DSC schemes when encoding Markov sources ($P_{t}=0.6$) with various *N* at a fixed code rate of 0.5 bit/symbol.

Figure 6 and Figure 7 show the decoding performance of SPDCAC for different sources with different *N* under a fixed code rate $R_{X}=0.5$ b/s. If we compare the results in Figure 6 with the results in Figure 7, we can find that SPDCAC can more easily achieve a better BER performance when decoding a source with a stronger internal correlation strength. This is because the posterior probability calculation method proposed in this paper can efficiently make use of the correlation among adjacent symbols in the source sequence to improve the decoding performance. Compared with DJSCAC, SPDCAC has the same or better BER performance under all H(X|Y), which reflects that SPDCAC can better utilize the internal correlation of the source to improve the decoding performance than DJSCAC.

**Figure 8:**
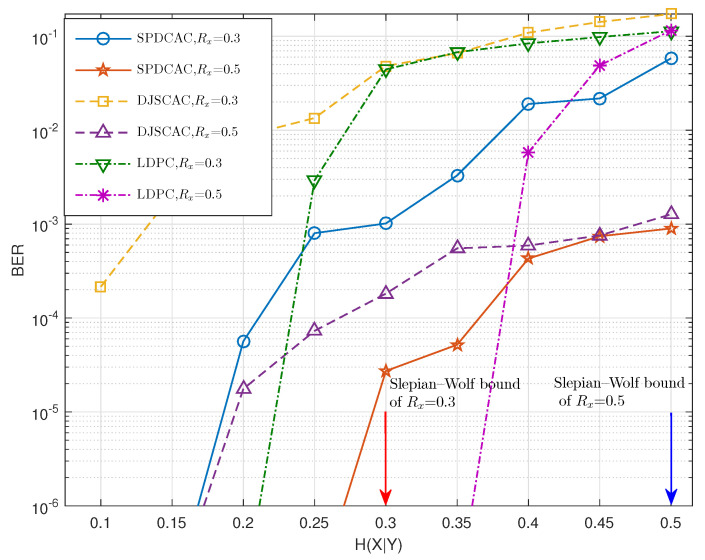
BER performances of different DSC schemes when encoding a Markov source ($P_{t}=0.88$) with N=1024 at various code rates.

Figure 8 shows the decoding performance of different schemes when coding the same source at different code rates. It can be seen that in some code rate cases, SPDCAC can exhibits a better BER performance than that of DJSCAC. Compared with the DSC scheme based on the LDPC code, SPDCAC exhibits a better BER performance when H(X|Y) is larger, and this advantage is more significant when the code rate is lower.

The decoding performance of a LDPC code-based DSC scheme is related to the number of parity bits (or the block size). Therefore, when the length of the source sequence is large, and H(X|Y) is small, the decoder can easily become lossless when decoding results. However, the BER performance of the DSC scheme based on LDPC codes will deteriorate rapidly as H(X|Y) increases. This shows that it is difficult for an LDPC code-based DSC scheme to ensure a better decoding performance when the correlation strength between *X* and *Y* is weak. Although the DSC scheme based on the LDPC code will ensure a better decoding performance when coding the source with weaker internal correlation strength, in the case of coding a source with strong internal correlation strength, the BER performance of the DSC scheme based on the LDPC code can only be better than that of SPDCAC when *N* is large and H(X|Y) is small. However, when the internal correlation strength of the source is strong and *N* is small, the decoding performance of the DSC scheme based on the LDPC code is completely inferior to that of SPDCAC.

### 4.4. Coding Complexity

The decoding complexity of DAC increases with the maximum width *M* of the decoding tree, and the encoding complexity of DAC is linear, like a classical AC [9]. In the SPDCAC encoding process, 1/n of the total number of the source symbols are purged from the source sequence, and the remaining source symbols are encoded using a conditional AC. Therefore, the encoding complexity of SPDCAC is proportional to $O(\frac{n-1}{n}N)$, which is slightly lower than that of DAC and DJSCAC. The decoding tree construction method of SPDCAC is the same as that of DAC, so the computational complexity of the decoding algorithm is the same as that of DAC and DJSCAC, which is proportional to O(MN). However, in the actual decoding process, decoding ambiguity will simultaneously occur in all decoding paths in the proposed scheme, while the decoding ambiguity of each decoding path in the DAC and DJSCAC appears randomly. Therefore, the computational complexity of the decoding algorithm in the proposed scheme will be slightly higher than those of the DAC and DJSCAC.

Table 3 shows the average encoding and decoding times (seconds) for SPDCAC and DJSCAC under different lengths and different maximum decoding tree widths (code rate = 0.5b/s, H(X|Y)=0.25, $P_{t}=0.8$). SPDCAC and DJSCAC are running on a personal computer equipped with an Intel Core i7-6700HQ 2.60 GHz processor.

From Table 3, it can be seen that the actual decoding times of SPDCAC and DJSCAC will accordingly grow as the maximum width of the decoding tree and the sequence length are increased. The actual decoding time of SPDCAC is about 1.2 times that of DJSCAC, which means that the decoding complexity of SPDCAC and DJSCAC is the same order of magnitude. Although the actual decoding time of SPDCAC is longer than that of DJSCAC, the actual encoding time of SPDCAC is shorter than that of DJSCAC. This is because SPDCAC reduces the number of source symbols that need to be encoded by purging the source sequence. This can reflect the characteristics that SPDCAC meets the low energy consumption requirements of the DSC coding scheme for the encoder.

## 5. Conclusions

A new DSC implementation named source symbol purging-based distributed conditional arithmetic coding is proposed. At the encoder of this scheme, some source symbols are purged from the source sequence to obtain additional compression gain, and a forbidden symbol is added to the source alphabet to ensure a better bit error rate performance at the decoder. The main feature of this scheme is that the purged symbols still have to be used as the context of the subsequent symbols to be encoded, so the decoder can utilize the correlation between the adjacent symbols in the source sequence to improve the decoding performance. Based on this feature, an improved calculation method for the posterior probability is proposed for the decoding process. The simulation results show that SPDCAC exhibits a better coding performance and encoding complexity than the DSC scheme based on expanding probability intervals. Compared with some source and channel code-based distributed source coding schemes, the proposed algorithm exhibits a better performance under the same compression ratio, especially when the internal correlation strength of the source sequence is strong. Therefore, SPDCAC is very suitable for application scenarios such as distributed video coding and wireless sensor networks. A limitation of this study is that the purging rate is fixed. This scheme should be extended to the adaptive purging rate in the future.

## Figures and Tables

**Table 1:** Transition probability matrix.

Transition Probability	$X_{i}=0$	$X_{i}=1$
**$X_{i-1}=0$**	$P_{t}$	$1-P_{t}$
**$X_{i-1}=1$**	$1-P_{t}$	$P_{t}$

**Table 2:** The RMCR of SPDCAC and DJSCAC when coding different sources.

Encoding Approach	RMCR (Bit/Symbol)			
	$P_{t}=0.9$	$P_{t}=0.8$	$P_{t}=0.7$	$P_{t}=0.6$
AC	0.4844	0.734	0.898	0.962
DJSCAC	0.3437	0.4687	0.4765	0.4922
SPDCAC	0.2422	0.3828	0.4238	0.5

**Table 3:** The average encoding and decoding time (s) of SPDCAC and DJSCAC.

*N* and *M* Size	Average Encoding Time (s)		Average Decoding Time (s)	
	SPDCAC	DJSCAC	SPDCAC	DJSCAC
N=128	0.00138	0.00143	0.059	0.046
M=128				
N=128			0.12	0.098
M=256				
N=1024	0.0042	0.0045	0.43	0.39
M=128				
N=1024			1.11	0.91
M=256				
